# Ochratoxin A Producing Fungi, Biosynthetic Pathway and Regulatory Mechanisms

**DOI:** 10.3390/toxins8030083

**Published:** 2016-03-21

**Authors:** Yan Wang, Liuqing Wang, Fei Liu, Qi Wang, Jonathan Nimal Selvaraj, Fuguo Xing, Yueju Zhao, Yang Liu

**Affiliations:** 1 Institute of Food Science and Technology, Chinese Academy of Agricultural Sciences, 1 Nongda South Road, Xibeiwang Town, Haidian District, Beijing 100193, China; wangyan062006@163.com (Y.W.); wanglq20082009@163.com (L.W.); liufei0823@yeah.net (F.L.); juwubawangqi@163.com (Q.W.); sjonnim@gmail.com (J.N.S.); fgxing@163.com (F.X.); zhaoyueju@caas.cn (Y.Z.); 2 Key Laboratory of Agro-products Processing, Ministry of Agriculture, 1 Nongda South Road, Xibeiwang Town, Haidian District, Beijing 100193, China

**Keywords:** ochratoxin A, producing fungi, biosynthetic pathway, regulatory mechanisms

## Abstract

Ochratoxin A (OTA), mainly produced by *Aspergillus* and *Penicillum* species, is one of the most important mycotoxin contaminants in agricultural products. It is detrimental to human health because of its nephrotoxicity, hepatotoxicity, carcinogenicity, teratogenicity, and immunosuppression. OTA structurally consists of adihydrocoumarin moiety linked with l-phenylalanine via an amide bond. OTA biosynthesis has been putatively hypothesized, although several contradictions exist on some processes of the biosynthetic pathway. We discuss recent information on molecular studies of OTA biosynthesis despite insufficient genetic background in detail. Accordingly, genetic regulation has also been explored with regard to the interaction between the regulators and the environmental factors. In this review, we focus on three aspects of OTA: OTA-producing strains, OTA biosynthetic pathway and the regulation mechanisms of OTA production. This can pave the way to assist in protecting food and feed from OTA contamination by understanding OTA biosynthetic pathway and regulatory mechanisms.

## 1. Ochratoxin A

Ochratoxin A (OTA) was first isolated as a secondary metabolite from *Aspergillus ochraceus* in 1965 [1]. OTA was identified as a toxic metabolite and considered one of the most important mycotoxins. OTA has a significant economic impact on food commodities in that OTA producing fungi were found to be a contaminant in a wide variety of foodstuffs. OTA is mainly produced by some *Aspergillus* and *Penicillium* species. On analyzing OTA structure, it is found as a polyketide-derived secondary metabolite and contains a dihydrocoumarin moiety coupled to an l-β-phenylalanine (Phe), derived from the shikimic acid pathway, by an amide bond. Its chemical name is: l-phenylalanine-*N*-[(5-chloro-3,4-dihydro-8-hydroxy-3-methyl-1-oxo-1H-2-benzopyrane-7-yl)carbonyl]-(*R*)-isocoumarin [2].

OTA is a potent nephrotoxic mycotoxin in nature and also displays other adverse effects such as hepatotoxicity, teratogenicity, and immunosuppression [3]. OTA has been proven to be carcinogenic in kidney and liver [4]. It has been classified as a group 2B human carcinogen by the International Agency for Research on Cancer (IARC), and World Health Organization (WHO) [5]. OTA has been putatively implicated in the etiology of Balkan endemic nephropathy (BEN) and recognized to be related to urinary-tract tumors [4]. Because of the hazard posed by this mycotoxin, the amount of OTA permitted in food is regulated by European Union and Codex [6].

Here, the OTA producing fungi, its biosynthetic pathway, and its regulation mechanisms will be summarized. All these aspects contribute to understanding OTA biosynthesis and regulation. This assists in realizing the correlation between environmental factors and OTA production and protecting food and feed from the fungal contamination and OTA production.

## 2. Ochratoxin A Producing Fungi

OTA is mainly produced by some species in *Aspergillus* and *Penicillium*. OTA presence in various food commodities such as cereals, beans, spices, dried fruits, nuts and oilseeds has been widely reported around the world. In recent years, several studies reported the production of OTA by other fungal species, especially in *Aspergillus* and *Penicillium* species (Table 1). They contaminate different crops with a different distribution depending on climatic conditions. *Aspergillus* species predominate in warm and temperate regions while *Penicillium* isolates are frequent in colder areas [7]. OTA production is higher at 0.98 *a*_w_, regardless of the temperature level, but its production tends to rise at the optimum temperature, between 25 and 30 °C in *A. ochraceus* [7].

Among the OTA-producing *Aspergillus* species, most of them belong to two sections: *Circumdati* (*A. ochraceus* group) and *Nigri* (*A. carbonarius* and *A. niger*) [8]. Earlier, *A. ochraceus* was considered to be the main source for OTA production in relatively warmer regions, while *P. verrucosum* being in colder areas [9]. However, *A. westerdijkiae*, a potential OTA producing fungus that is phylogenetically similar to *A. ochraceus*, is frequently found in tropical regions, and although it does not grow at 37 °C, it is still considered to be one of the important OTA producers [10].

Varga *et al.* detected OTA production in different *Aspergillus* species, *i.e.*, *A. alliaceus*, *A. sclerotiorum*, *A. sulphureus*, *A. albertensis*, *A. auricomus*, and *A. wentii* [11]. Among them, *A. alliaceus* is an important OTA producing fungus contaminating figs in California [12]. Reports by Rizzo *et al.* indicated that other species of *Aspergillus*, such as *A. auricomus*, *A. fumigates*, *A. versicolor*, *A. albertensis* and *A. wentii*, could also produce OTA [13]. Another study reported the production of OTA by *A. niger*, *A. lacticoffeatus* and *A. sclerotioniger* [14]. *A. cretensis*, *A. flocculosus*, *A. pseudoelegans*, *A. roseoglobulosus*, *A. westerdijkiae*, *A. sulphurous*, and *Neopetromyces muricatus* are consistently reported to be the large producers of OTA [10]. *A. steynii*, which contaminates coffee, appeared to be an OTA producer [15,16]. *A. welwitschiae* (formerly *A. awamori*) was also confirmed as an OTA producer [17,18]. Recently, in submerge driparian decomposing leaves, a new OTA producing fungus was identified, *A. affinis* [19].

OTA-producing *Penicillium* species like *P. verrucosum*, *P. nordicum* and *P. expansum*, have been isolated and identified [8,20]. *P. nordicum* generally contaminates food rich in NaCl and protein, such as cheeses and dry cured meats, while *P. verrucosum* usually contaminates cereals and is occasionally found on dry cured ham and brined olives [21]. Other *Penicillium* species, *P. chrysogenum*, *P. glycyrrhizacola* and *P. polonicum*, were able to synthesize OTA on fresh or dry liquorice where *P. chrysogenum* was a high OTA producer [22]. Some *Penicillium* species like *P. brevicompactum*, *P. crustosum*, *P. olsonii* and *P. oxalicum* produced much lower levels of OTA, less than 0.1 ppb [23]. The only well ascertained OTA producers in *Penicillium* genus are *P. verrucosum* and *P. nordicum*, although there are many reports on other OTA-producing *Penicillium* species as others may erroneously be claimed to be OTA producers or are sometimes erroneously identified.

## 3. Ochratoxin A Biosynthetic Pathway

### 3.1. OTA Related Metabolites

l-Phenylalanine was recognized a precursor of OTA by radiolabeling experiments where 1-^14^C-phenylalanine was incorporated into the phenylalanine moiety when supplied in the culture of *A. ochraceus* [1,24]. The isocoumarin moiety of OTA was mostly derived via acetate condensation with the addition of 2-^14^C-Na-acetate.

The biosynthesis of ^14^C-labeled OTA was carried out in the liquid culture of *P. verrucosum* sp. 1761 using 2-^14^C-Na-acetate and 2-^14^C-malonic acid as precursors and they were both found to be incorporated in OTA biosynthesis [25]. Furthermore, the whole molecular structure of OTA was labeled with 2-^14^C-Na-acetate as precursor. However, the isocoumarin moiety of OTA was exclusively labeled by 2-^14^C-malonic acid, indicating that both acetate and malonic acid are the precursors of OTA biosynthesis. Malonic acid could be involved in the isocoumarin moiety biosynthesis but not the phenylalanine moiety biosynthesis, whereas acetate was transformed to both the phenylalanine moiety and the isocoumarin portion as a precursor.

^14^C-labeled precursor feeding experiments demonstrated that the carboxyl carbon of the amide group was derived from methionine at C-7 in the isocoumarin moiety of OTA [24]. The methylation inhibitor ethionine completely inhibited OTA production [26], which further confirmed that methionine was the substrate as the donor of carboxyl carbon at C-7 of the isocoumarin moiety. Ochratoxin α (OTα) was the dihydroisocoumarin derivative with a carboxyl group at C-7. OTA was synthesized by the linking bond between OTα and phenylalanine by a crude cell-free enzyme preparation [27]. Corresponding des-chloro analogues were identified as ochratoxins B (OTB) and ochratoxin β (OTβ) [28]. OTβ and mellein had been isolated from ochratoxinogenic *A. ochraceus* cultures [29,30], while OTα was not detected in OTA related intermediate at the beginning of *A. ochraceus* growth, which was actually obtained by acid hydrolysis of OTA [24,31,32,33]. However, Gallo *et al.* reported that OTα could be detected in liquid cultures of *A. carbonarius* ITEM 5010 analyzed by HPLC-FLD and HPLC-HRMS [34]. The incorporation of ^36^Cl into the isocoumarin moiety of OTA was demonstrated by Wei *et al.* [35].

Mellein and 4-hydroxymellein could be produced and isolated by an OTA-producing strain of *A. ochraceus* [29]. Their structures are similar to the dihydroisocoumarin portion of ochratoxin A and B. The lactoneacids from ochratoxin A and B were structurally related to mellein and probably related to mellein genetically [2].

### 3.2. OTA Biosynthesis Genes

#### 3.2.1. Polyketide Synthase (PKS) and Non-Ribosomal Peptide Synthetase (NRPS)

PKS and NRPS are large multimodular enzymes involved in biosynthesis of polyketide and peptide products as secondary metabolites, respectively [36]. From the structure of OTA, its dihydrocoumarin moiety consists of a polyketide that is believed to be catalyzed by PKS. The polyketide moiety is linked with l-phenylalanine through the carboxyl group catalyzed by a peptide synthase, generally NRPS [37]. The hybrid PKS-NRPS occurs frequently in the production of secondary metabolites [36].

Analysis of differentially expressed genes was carried out under different conditions that were either suitable for OTA production or not by Differential Display Reverse Transcriptase-PCR (DDRT-PCR) [38,39]. The differentially expressed genes contained putative genes encoding PKS, NRPS and halogenase involved in OTA biosynthesis [39]. A putative gene cluster was then identified in *P. nordicum*, which was responsible for OTA biosynthesis [40,41,42]. A polyketide synthase (*otapksPN*) and a non-ribosomal peptide synthetase (*npsPN*) were putatively involved in OTA biosynthetic pathway [40,41]*.* This was further confirmed at gene transcriptional level under OTA producing conditions and the mutant analysis via gene inactivation. Interestingly, the *pks* and *nrps* homolog genes could have not been identified in ochratoxigenic *Aspergillus* species. This indicates that the ochratoxin PKS and NRPS might be genus specific between *Penicillium* and *Aspergillus* species.

From then on, *pks* genes required for OTA biosynthesis were, respectively, identified by gene inactivation and OTA analysis in *A. ochraceus* [43], *A. westerdijkiae* [44], *A. carbonarius* [45] and *P. verrucosum* [46]. A *pks* gene, *AcOTApks*, involved in OTA biosynthesis was identified and recognized to play a critical role in the OTA biosynthesis in *A. carbonarius*, and then it was further confirmed that a *nrps* gene, located closer to the *pks*, played an important role in OTA biosynthesis by the *nrps* deletion with no OTA production [34]. The amino acid sequences of these PKS and NRPS showed high similarity, compared to the predicted OTA gene cluster in *A. niger* CBS513.88 [47].

#### 3.2.2. Halogenase and P450 Oxidase

Geisen *et al.* identified the putative gene cluster responsible for the OTA biosynthesis and among these genes in this cluster, there is an open reading frame encoding a putative OTA biosynthetic protein homologous to a halogenase/chloroperoxidase responsible for the introduction of the chlorine atom by a chlorination step in OTA biosynthetic pathway [39,42].

Actually, it is also demonstrated that an oxidase may play an important role in the step of oxidized reaction during the biosynthetic process of the final polyketide precursor. A gene encoding a cytochrome P450 family protein was identified by analyzing differentially expressed genes between ochratoxin-producing and non-producing strains of *A. westerdijkiae* [48]. Among differentially expressed genes, three putative oxidoreductase genes were highly up-regulated in the OTA producing strain compared to non-producing strain.

### 3.3. Putative OTA Biosynthetic Pathway

Although OTA is a very important mycotoxin for food security and human health, the genetic background of OTA biosynthesis is little known compared to the other important mycotoxins such as aflatoxins, fumonisins and zearalenones [37].

It was likely considered that the biosynthesis of the lactone acids part of ochratoxin A and B were derived from head-to-tail condensation of five acetate (or one acetate and four malonate) units by an analogy of the biosynthesis of citrinine in *A. candidus* [49] and oospolactone in *Oosporu ustringenes* [50] according to Steyn and Holzapfel [24].

OTA biosynthetic pathway had been proposed as a scheme by Huff and Hamilton [51]. They hypothesized that mellein, catalyzed by PKS, was oxidized to OTβ and then transformed to OTα by a halogenase/chloroperoxidase. Subsequently, OTα was esterified to ochratoxin C via link with the ethyl ester, and finally biosynthesized to OTA by a deesterification reaction.

However, they ignored the putatively ubiquitous intermediate, OTB, in some processes of OTA biosynthetic pathway. During the study on *pks* gene involved in OTA biosynthesis in *A. westerdijkiae* NRRL 3174, it was found that mellein played no role in OTA biosynthesis on analyzing the secondary metabolites [44,52]. This was further supported by ^14^C-labelled precursor feeding experiments, which did not support the intermediary role of mellein confirmed by Harris and Mantle [31]. On the other hand, the ester ochratoxin C, proposed to protect the phenylalanine carboxyl by Huff and Hamilton during the last OTA biosynthetic step [51], was found to have no role as the intermediate. In Harris and Mantle’s study, they proposed that OTβ to OTα is catalyzed by a halogenase/chloroperoxidase and eventually to OTA via an amide bond with phenylalanine [31]. Chlorination of OTα probably preceded the biotransformation from OTα to OTA. This indicated that chlorination was a penultimate biosynthetic step in OTA biosynthesis. Moreover, they proposed an alternative pathway on account of the role of OTB in which OTA was transformed through the synthetic step from OTβ to OTB via an amide group, but this did not explain the role of OTα involvement in OTA biosynthesis. It seems to support that OTB was not a byproduct of OTA because the levels of OTA and OTB produced by *A. ochraceus* differed from one carbon or nitrogen source to another [53]. If this view were considered correct, both OTA and OTB would be affected and changed in a similar way as expected by the different nutrition conditions.

Similarly, ochratoxin C was not considered to be involved in OTA biosynthesis by Gallo *et al.* as it was not detected in *A. carbonarius* liquid culture [34]. They represented a novel OTA biosynthetic pathway similar to the alternative pathway of Harris and Mantle, *i.e.*, OTβ to OTB by NRPS, and then to OTA by a halogenase/chloroperoxidase. However, OTα was mostly likely to be derived from the biodegradation activity in *A. carbonarius* ITEM 5010, because OTA was quickly and completely biotransformed and accordingly OTα was accumulated at a similar time during the culture of *A. carbonarius*, by adding with exogenous OTA in the mutant of *nrps* gene disruption. This might be a self-protection strategy in this fungus.

## 4. Regulation Mechanisms of Ochratoxin A Biosynthesis

### 4.1. Specific Regulators of OTA Pathway

Generally, among mycotoxins gene cluster, there exists one or more regulatory genes controlling the expression of biosynthetic genes. In aflatoxin biosynthetic gene cluster, *aflR* positively regulated aflatoxin and sterigmatocystin synthesis by binding to the promoter region of all the biosynthetic genes except *aflJ* and activating their transcription [54,55]. The gene cluster of fumonisin biosynthesis also contains a transcription factor, FUM21, predicted to be a Zn(II)-2Cys6 DNA-binding protein [56]. FUM21 positively activates *FUM* gene transcription and plays an important role in fumonisin biosynthesis. Among the zearalenone biosynthetic cluster genes, *ZEB2* encode a transcriptional factor and expressed two isoforms (ZEB2L and ZEB2S) via an alternative promoter in *F. graminearum* [57]. ZEB2L, a basic leucinezipper (bZIP) DNA-binding domain at the N-terminus included, interacts with ZEB2S, lacking the bZIP domain, to generate a formation of a heterodimer that regulates zearalenone biosynthesis. Among *Tri* genes in trichothecene biosynthetic gene cluster, two *Tri* genes, *Tri6* and *Tri10*, regulate T-2 toxin production [58]. *Tri10* inactivation could inhibit T-2 biosynthesis which markedly decreased the expression profile of all other *Tri* genes [59,60]. *Tri6*, encoding a Cys_2_His_2_ zinc finger transcription factor, was involved in T-2 production by positively regulating the transcription levels of *Tri5* and *Tri4* [61] and so was the putative patulin regulatory gene *patL* in patulin biosynthetic gene cluster controlling patulin production [62]. Overall, one or more biosynthetic pathway-specific regulators are located in many mycotoxin biosynthetic gene clusters, control the expression of their biosynthetic genes and affect the respective mycotoxin production. Therefore, there may be a pathway-specific regulatory gene controlling OTA production by regulation the expression profile of OTA biosynthetic genes.

A putative *pks* gene *aolc35-12* could code for a certain polyketide compound complementary for the expression of *aoks1*, which might be required for OTA biosynthesis in *A. westerdijkiae* NRRL 3174 [44], which activated OTA biosynthesis in *A. westerdijkiae* [52]. The inner mechanisms have not yet been confirmed whether the polyketide product could play the complementary role for the *pks* gene to involve in OTA biosynthesis. Similarly, *AcOTApks*, characterized as a component of the OTA biosynthetic pathway [45], showed difference from an ACpks protein suggesting its likely involvement in OTA biosynthesis in *A. carbonarius* [63]. There also exists two PKSs involved in OTA biosynthesis likely by different manners [43]. Interestingly, for some instances that other secondary metabolites in fungi likely requiring two PKSs for a polyketide production, such as asperfuranone in *A. nidulans* [64], zearalenone in *Gibberella zeae* [65,66], T-toxin in *Cochliobolus heterostrophus* [67], compactin in *Penicillium citrinum* [68], and lovastatin in *A. terreus* [69,70]. However, the mechanisms of these two different PKSs that are likely responsible for OTA biosynthesis still remain to be established in future.

### 4.2. General Regulatory Pathways for OTA Biosynthesis

#### 4.2.1. Velvet Complex Controls OTA Production

It was identified that the heterotrimeric velvet complex, VelB/VeA/LaeA, could coordinate fungal development with secondary metabolism in *A. nidulans*, in response to light [71]. Among velvet complex, VeA subcellular localization depends on light [72]. Interestingly, light is a critical influence on growth and OTA production. Conidia formation, mycelial growth and OTA production could be affected by white and UV light treatment in *A. ochraceus* [73]. The inhibitory effects were strongest on growth and OTA production under red (long wavelength at 627 nm) and blue (short wavelength at 470–455 nm) light condition [74]. Fanelli *et al.* had similar results on growth, conidiation and OTA biosynthesis under the influence of light in *A. niger* [75]. Furthermore, VeA and LaeA transcriptional factors were studied via gene replacement strategy and found that inactivation of *veA* and *laeA* showed slighter differences in vegetative growth but a marked reduction in conidial production in *A. carbonarius* [76]. It was revealed that OTA production was promoted in *A. carbonarius* under dark conditions, whereas conidiation was activated with light treatment. OTA and conidial production was drastically decreased in Δ*veA* or Δ*laeA* null mutants. VeA was reported to be mainly located in the cytoplasm in response to light, while in the dark VeA was migrated into the cell nucleus in *A. nidulans* [72]. A putative nuclear localization signal (NLS) motif in the VeA amino acid sequence played an important role in the migration of VeA from the cytoplasm to the nucleus in *A. nidulans*. Accordingly, NLS motif was found in the *N*-terminal region of VeA in *A. carbonarius* putatively required for VeA to enter into the nuclei [76]. LaeA was reported to act chromatin-based regulation of secondary metabolites by modifications of histones acetylation [77]. Hence, it might be shown that VeA could be transported to the nucleus and interacted with LaeA under dark condition in *A. carbonarius* [76]. In this study, transcription level of *veA* and *laeA* was relatively similar in *A. carbonarius* in response to both light and dark, which led to the prediction that the subcellular localization of VeA was critical to the regulation of OTA production under light treatment, rather than the transcription level of the regulators [76]. Moreover, VeA and LaeA regulation might function at multiple layers of regulation network, such as transcription, post-transcriptional processing, translation, or posttranslational modification.

#### 4.2.2. Oxidative Stress

The correct redox balance is one of the most crucial factors for the regulation of the growth, conidial formation, and secondary metabolite biosynthesis in fungi. There are inner correlations between the modulation of oxidative stress and mycotoxin biosynthesis, such OTA or aflatoxin modulated by oxidative stress related transcription factor, Yap1 [78,79]. Reverberi *et al.* revealed that OTA production was highly correlated with lipoperoxidation. The inactivation of a lipoxygenase (LOX) gene (*AoloxA*) displayed different phenotype on colony morphology, conidia formation, and sclerotia production, a lower LOX activity, the diminished level of some oxylipins derived from linoleic acid, and remarkably inhibited OTA production in *A. ochraceus* [80]. To investigate the role of oxidative stress related transcription factors in regulating mycotoxin production and reactive oxygen species (ROS) produced under oxidative stress condition, they studied the role of oxidative stress related transcription factor, Ao*yap1*, a homolog of the *Yap-1* from yeast correspondingly [79]. The Ao*yap1* disrupted strain could not scavenge ROS efficiently as it remained at high level, which was proposed to be associated with the reduced activity of superoxide dismutases and catalases in that their gene expressions were obviously decreased overall in ∆Ao*yap1*, compared to the wild type. Moreover, the conidia formation was significantly reduced and the conidia tend to be larger in solid media in the mutant. Its growth was also reduced and delayed in ∆Ao*yap1* in response to CCl_4_. Interestingly, Geisen *et al.* reported that high oxidative stress conditions led to the changes of secondary metabolites from OTA to citrinin and there existed the cross-regulation of the ochratoxin A/citrinin in response to different environment factors [81]. In addition, Stoll *et al.* studied the differentially expressed proteins of *P. verrucosum* under light of short wavelength (450 nm) or in the dark using comparative proteome analysis and found that most of these proteins (light *vs.* dark) are assumed to be related to stress and general metabolic processes in the 46 significantly differential proteins identified. The OTA production is strongly reduced under light of short wavelength as the induction of stress-related proteins could efficiently normalize oxidative stress although light of short wavelength could lead to oxidative stress

#### 4.2.3. pH Regulates OTA Biosynthesis via PacC

External pH is regarded as a critical element to affect the growth, the development and secondary metabolites biosynthesis of fungi. Molds must adapt to their pH surroundings for their growth and reproduction. Many fungi can grow well in a broader pH range and the expression of certain genes are bound to the environmental pH [82]. Gene expression is efficiently regulated by transcriptional factors like PacC to produce several secondary metabolites by ambient pH. PacC contains three zinc fingers motifs binding to the consensus sequence “5′-GCCARG” in the promoter region in *A. nidulans* [83,84]. The transcriptional factor PacC actively inhibits known acid-expressed gene expression and enables the expression of known alkaline-expressed genes in *A. nidulans*. PacC regulated aflatoxin and sterigmatocystin production in *A. nidulans* and *A. Parasiticus*, respectively [85]. Kapetanakou *et al.* evaluated the effect of OTA production with the combined environmental factors, pH, water activity and temperature [86]. In their study, ambient pH seemed to have little specific effect compared to the influence of water activity for OTA biosynthesis in *A. ochraceus* and *A. carbonarius*. Esteban *et al.* found that OTA-producing *A. niger* not only adapted to a wider pH range for growth, but also produced OTA under a wider pH range [87]. Passamani *et al.* also reported that *A. carbonarius* and *A. niger* could grow and produce OTA well under a wider pH range [88]. They grew at the optimal condition with pH about between 5.0 and 6.5, and highest level of OTA production was observed at pH 5.35. Although several reviews have detailed the pH regulatory mechanism in *A. nidulans* [82,89,90,91], little is known about the cellular mechanisms of PacC regulating OTA production.

#### 4.2.4. Nutrient Sources on OTA Production via CreA and AreA

Nutrients availability is generally regulated for adaption to environmental factors in fungi. Medina *et al.* found that OTA production was significantly different under various carbon sources in three isolates of *Aspergillus* spp. from grape and generally the correlation of the OTA level and the content of carbon sources was actually positive [92]. However, no marked difference in the OTA production with regard to nitrogen source except that phenylalanine seemed to be favorable for the toxin production. In the other report of Medina *et al.*, they detected the capacity of OTA production using Yeast Extract Sucrose (YES) medium supplemented with 0.5% peptone but found no significant differences [93]. Moreover, bee pollen was regarded as a strong stimulator for OTA production as a substrate in *A. ochraceus*. Furthermore, OTA level was positively related to the concentration of bee pollen adding to YES medium. On the contrary, in another study on the influence of carbon and nitrogen resources for OTA production in *A. ochraceus*, they indicated similar results that different carbon sources positively influence OTA production in MCB medium, and different carbon sources repressed OTA production in OTA permissive PDY medium except lactose, which showed high induction of OTA biosynthesis not only in MCB medium but also in PDY medium [53]. In addition, the inorganic nitrogen source, ammonium chloride, strongly reduced OTA level, while organic nitrogen sources promoted OTA production. Interestingly, OTA production showed positively correlation with carbon and nitrogen sources, except urea [94].

Carbon and nitrogen sources, as main nutrient sources, were regarded to be mediated by transcriptional factors. MIG1 was identified as a zinc finger protein involved in the repression of glucose-regulated genes [95]. CreA/Cre1 proteins, homologous to MIG1, have been described in *A. nidulans* [96], *F. oxysporum* [97], *Gibberella fujikuroi* [98], and *Trichoderma reesei* [99]. CreA binding site (-SYGGRG) was identified by DNase I footprinting in *A. nidulans*, which was further confirmed by deletion of the CreA binding sites resulting in derepression of prolineutilization cluster gene [96,100]. Nitrogen metabolism was defined and confirmed by the GATA transcription factor AreA in *A. nidulans* [101,102]. The regulation of nitrogen catabolism has been described in detail as a complex network with several transcriptional factors, AreA, NmrA and MeaB, in *A. nidulans* [102]. Therefore, the regulation of nutrient sources on OTA production needs in-depth study.

#### 4.2.5. Osmotic Stress

Several signaling cascade pathways have been described as playing an important role for mycotoxin production by activating biosynthetic genes [71,82,103,104,105].

Increasing the amount of NaCl, leading to high osmotic stress, it was assumed that a HOG-like cascade (high osmolarity glycerol) was incorporated with signal transduction [106]. The correlations between the growth and OTA production were evaluated in adaptation to different concentrations of NaCl in *P. nordicum* and *P. verrucosum* [21]. *P. nordicum* grew well and produced higher level of OTA under different NaCl concentrations as this fungus occurred mainly in dry cured meats or cheeses which are highly rich in NaCl [107,108]. However, secondary metabolites shifted from citrinin to OTA in *P. verrucosum* by increasing concentrations of NaCl. It was proposed that the induction of OTA production under NaCl condition was related to the phosphorylation status of the HOG-MAP kinase [109]. Conversely, OTA production was decreased with the increased NaCl concentrations, but the phosphorylation level of HOG was increased in *A. carbonarius*. In addition, the growth of this fungus was also remarkably repressed under this condition. However, less impact on the growth and phosphorylation of HOG were observed with respect to high glucose concentrations, although the toxin production decreased with the increasing glucose concentrations. Moreover, to confirm whether HOG was indeed necessary for OTA production under high osmolar stress, *hog* gene disruption was carried out in *P. verrucosum* and the results demonstrated the regulation of OTA biosynthesis by the HOG-MAP kinase. In addition, they analyzed the correlation between the growth rate, the level of HOG phosphorylation and the mycotoxin biosynthesis under different NaCl concentrations in *P. verrucosum* with different ability to produce the mycotoxin [110]. It was demonstrated that a weak OTA producer showed a poor growth rate and the strongest phosphorylation level of HOG1 protein from 0 g/L NaCl to 50 g/L. Although it appeared to confirm the correlation between HOG and OTA production, the reason why the differences exist among OTA-producing strains or non-producing strains under high osmotic stress and the question of whether the different mechanisms are involved in regulating OTA production with signal transduction need further investigation.

## 5. Conclusions

OTA has been successively studied for about half a century from the beginning of its isolation from *A. ochraceus* [1]. It has been realized that OTA is highly harmful for human heath due to its nephrotoxicity, hepatotoxicity, carcinogenicity, teratogenicity, and immunosuppression [2,111]. Studies have indicated several enzymes, such as PKS, NRPS, halogenase and P450 oxidase, that responsible for some steps of OTA biosynthesis. From OTA structure and biosynthetic genes of these enzymes, the OTA biosynthetic pathway has been putatively proposed [31,34,51], but still some contradictions appear exist. Accordingly, genetic regulation has also been investigated with regard to the interaction with environmental factors. Recently, genomic resources of OTA producing strains have been obtained and analyzed by genome-wide sequencing technologies, such as for *A. nige*r CBS 513.88 and *A. carbonarius* ITEM5010 [45,47]. This will efficiently promote our understanding of the mechanisms of OTA production and regulation. Eventually, it will assist in developing effective strategies for protection food and feed from OTA contamination by understanding its biosynthesis and regulation.

## Figures and Tables

**Table 1:** Ochratoxin A production mainly by *Aspergillus* and *Penicillium* species.

Number	Organism/Name	SubGroup	Section	Location	Source
1	*Aspergillus affinis*	Ascomycetes	Circumdati	Italy	decomposingleaves, fluvial mycobiota
2	*Aspergillus albertensis*	Ascomycetes	Flavi	Canada	ear swab
3	*Aspergillus alliaceus*	Ascomycetes	Flavi	USA, Australia, Indonesia	macrobasis albida, great barrier reef, kemiri nut, soil
4	*Aspergillus welwitschiae*	Ascomycetes	Nigri	Japan, Portugal, Spain, Italy, Greece	koji, Grapes
5	*Aspergillus carbonarius*	Ascomycetes	Nigri	China, Italy, Australia, USA	grape, beer, coffee
6	*Aspergillus cretensis*	Ascomycetes	Circumdati	Greece, Israel	citrus, soil
7	*Aspergillus flocculosus*	Ascomycetes	Circumdati	Slovenia, India, Netherlands, Greece	saltern
8	*Aspergillus lacticoffeatus*	Ascomycetes	Nigri	Venezuela, Indonesia	coffee bean, soil
9	*Aspergillus niger*	Ascomycetes	Nigri	China, Italy, Spain, Germany, USA	grape, beer, cereal, coffee, triticum aestivum, zeamays
10	*Aspergillus ochraceus*	Ascomycetes	Circumdati	China, Italy, Portugal, Denmark, UK, Japan, France	cereal, coffee, beverage, grape, zeamays
11	*Aspergillus pseudoelegans*	Ascomycetes	Circumdati	Costa Rica	soil
12	*Aspergillus roseoglobulosus*	Ascomycetes	Circumdati	Bahamas	decaying leave of rhizophora mangle
13	*Aspergillus sclerotioniger*	Ascomycetes	Nigri	India	coffee bean, green coffee
14	*Aspergillus sclerotiorum*	Ascomycetes	Circumdati	USA, Thailand, China	malus sylvestris, fruit, soil
15	*Aspergillus steynii*	Ascomycetes	Circumdati	India, China, Australia, Panama, Argentina, Sri Lanka	green coffee bean, rice, arecha catechu, soybean
16	*Aspergillus sulphureus*	Ascomycetes	Circumdati	India, China	alkaline soil
17	*Aspergillus westerdijkiae*	Ascomycetes	Circumdati	South Africa, China, Slovenia, India	rice, beverage, green coffee bean, saltern, sorghum, corn, chili, anise, grapes
18	*Neopetromyces muricatus*	Ascomycetes	-	Australia, Philippines, Indonesia	peanut, soil
20	*Penicillium nordicum*	Ascomycetes	-	Germany, Italy	cheese, fermented meats
21	*Penicillium verrucosum*	Ascomycetes	-	Germany, Australia, Italy, UK, Sweden	cereal, grape, triticum durum, rye, barely
